# Effects of Peptide-Induced Immune Tolerance on Murine Lupus

**DOI:** 10.3389/fimmu.2021.662901

**Published:** 2021-05-19

**Authors:** Ram P. Singh, Bevra H. Hahn, David S. Bischoff

**Affiliations:** ^1^ Research Service, Veteran Administration Greater Los Angeles Healthcare System, Los Angeles, CA, United States; ^2^ Division of Rheumatology, Department of Medicine, University of California, Los Angeles, Los Angeles, CA, United States; ^3^ Department of Medicine, University of California, Los Angeles, Los Angeles, CA, United States

**Keywords:** regulatory B cells, immune tolerance and regulation, pConsensus peptide (pCons), systemic lupus erythematosus, Anti-DNA Ab, polymorphonuclear cells (PMNs), granulocytes

## Abstract

The regulation of autoimmunity and the molecular mechanisms by which different immune cells, including T cells, polymorphonuclear leukocytes (PMN-granulocytes), and B cells suppress autoimmune diseases is complex. We have shown previously that BWF1 lupus mice are protected from autoimmunity after *i.v.* injection or oral administration of tolerogenic doses of pCons, an artificial synthetic peptide based on sequences containing MHC class I and MHC class II determinants in the VH region of a J558-encoded BWF1 anti-DNA Ab. Several T cell subsets can transfer this tolerance. In this study, we determined the potential roles of granulocytes, B cells and regulatory T cells altered by pCons treatment in the BWF1 (NZB/NZW) mouse model of lupus. Immunophenotyping studies indicated that pCons treatment of BWF1 mice significantly increased CD4^+^FoxP3^+^ T cells, reduced the percent of B cells expressing CD19^+^CD5^+^ but increased the percent of CD19^+^CD1d^+^ regulatory B cells and increased the ability of the whole B cell population to suppress IgG anti-DNA production *in vitro*. pCons treatment significantly decreased the expression of CTLA-4 (cytotoxic T-lymphocyte-associated protein-4) in CD8^+^ T cells. In addition, peptide administration modified granulocytes so they became suppressive. We co-cultured sorted naïve B cells from mice making anti-DNA Ab (supported by addition of sorted naive CD4^+^ and CD8^+^ T cells from young auto-antibody-negative BWF1 mice) with sorted B cells or granulocytes from tolerized mice. Both tolerized granulocytes and tolerized B cells significantly suppressed the production of anti-DNA *in vitro*. In granulocytes from tolerized mice compared to saline-treated littermate controls, real-time PCR analysis indicated that expression of *interferon-induced TNFAIP2* increased more than 2-fold while *Ptdss2* and *GATA1* mRNA were up-regulated more than 10-fold. In contrast, expression of these genes was significantly down-regulated in tolerized B cells. Further, another IFN-induced protein, Bcl2, was reduced in tolerized B cells as determined by Western blot analyses. In contrast, expression of FoxP3 was significantly increased in tolerized B cells. Together, these data suggest that B cells and granulocytes are altered toward suppressive functions by *in vivo* tolerization of BWF1 mice with pCons and it is possible these cell types participate in the clinical benefits seen *in vivo*.

## Introduction

Regulatory B cells and regulatory polymorphonuclear leukocytes (PMNs-granulocytes) influence immunity but are not well understood in systemic autoimmunity. Lupus causes significant morbidity, mortality, and economic loss. Systemic lupus erythematosus (SLE) is probably initiated by autoantibodies (*e.g*. anti-DNA) and immune complexes that induced inflammation and organ damage (1). One of the organs affected in some patients with SLE is the kidney and lupus nephritis is a leading cause of end stage kidney disease and death. Approximately two million people suffer from this disease, with the majority of cases being women of childbearing age. Lupus is a gender-biased disease with a female to male ratio of 9:1. African-American women are three times more likely to get lupus than Caucasian women. Lupus is also more common in Hispanic, Asian, and Native American women than in Caucasians. In the last 50 years, there have been only two new lupus-specific therapy approved by the FDA, Benlysta (anti-BAFF, and voclosporin (a calcineurin inhibitor). Current treatments, including the newer ones, rarely induce sustained disease remission. Therefore, additional treatment strategies are urgently needed. The modulation of abnormal immune regulation is an object of intense investigation in several experimental autoimmune diseases with the goal to limit the numbers of abnormal pathogenic cells and autoantibodies, and to achieve restoration of immune system self-tolerance by the administration of peptides that induce regulatory cells.

We have focused studies in a mouse model of SLE, the BWF1-New Zealand Black/New Zealand White (NZB/NZW) mouse, which has several characteristics in common with human SLE (2, 3). These mice spontaneously develop fatal immune mediated glomerulonephritis with high titers of anti-nuclear antibodies including high affinity IgG antibodies to dsDNA and show female to male bias. In this model, we used a peptide, pCons, to induce regulatory cells which are protective in SLE. We studied gene expression in splenocytes using Affymetrix microarray analysis (448 genes were differentially regulated one week after tolerance induction), followed by validation studies with quantitative real-time RT-PCR in CD4^+^ and CD8^+^ T cell subsets. In the current study, we test for potential molecular mechanisms that govern the function of tolerized B cells (including B_regs_) and PMNs-granulocytes in this model.

Regulatory B cells (B_regs_), a novel subpopulation of B cells, are a significant area of research due to their therapeutic relevance, immune regulatory function, and ability to sustain self-tolerance (4–6). Evidence suggesting a role for (B_regs_) in the immune system has been described since the 1970s. These studies suggest that there is a potential role for B_regs_ in reducing T cell activity and inducing immune tolerance (7–10). Over the past decade, B_regs_ have been identified in many autoimmune diseases (11–17). Approaches to manipulate B cells in a manner that is beneficial in attenuating inflammatory and autoimmune conditions, including SLE, are not clear. The mechanisms by which B_regs_ influence the functions of CD4^+^ and CD8^+^ T_regs_ are not known. Additionally, genes expressed in B_reg_ cells (other than IL-10) that offer protective responses and their molecular mechanisms of function remain to be defined.

Polymorphonuclear neutrophils (PMN-granulocytes) have been shown to play a role in a variety of autoimmune diseases including Rheumatoid Arthritis (18), Inflammatory Bowel Disease (19, 20), and Experimental Autoimmune Encephalomyelitis (EAE) (21). Studies have shown that PMNs-granulocytes are capable of interacting with T cells by presenting class I and class II restricted antigens on their surface (22–26) as well as in a non-MHC restricted fashion (22). PMNs-granulocytes have also been shown to express the costimulatory molecules CD80 and CD86 (27), the regulation of which is important in autoimmunity and immune tolerance. In patients with SLE, granulocytes undergoing NETosis are increased, and the nets contain DNA/nucleosome/proteins that promote autoreactivity and production of type 1 IFNs (28). The exact mechanisms of PMNs-granulocytes and B_regs_ interaction with other regulatory cells and their cross-talk are currently poorly defined. In this report, we provide novel evidence that pCons tolerance induces CD4^+^FoxP3^+^ T cells and potent regulatory B cells and granulocytes capable of suppressing autoimmunity *in vitro* in a murine model of SLE. Understanding the role of regulatory T cells, B cells and granulocytes may provide novel mechanistic insight for SLE and expand our knowledge of immune tolerance and can identify potential new targets for SLE.

## Materials and Methods

### Mice

NZB (H-2d/d), NZW (H-2z/z) and NZB/NZW F1 (H-2d/z) mice were purchased from the Jackson Laboratories (Bar Harbor, ME, USA) or bred at the University of California Los Angeles (UCLA). All mice were treated in accordance with the guidelines of the University of California Los Angeles Animal Research Committee, an Institution accredited by the Association for Assessment and Accreditation of Laboratory Animal Care (AAALAC). Mice were housed in pathogen-free conditions. Female mice were used for all experiments.

### Peptides

The peptides used in this study and the MHC molecules they bind have been described earlier (29, 30). pCons (FIEWNKLRFRQGLEW), the artificial tolerizing peptide, contains T-cell determinants based on the J558 VH regions of several murine anti-dsDNA Ab from BWF1 mice (29, 31–35). Peptides were synthesized at Chiron Biochemicals (San Diego, CA, USA), purified to a single peak on high-performance liquid chromatography, and analyzed by mass spectroscopy for expected amino acid content.

### Treatment of Mice

Ten- to twelve-week-old BWF1 mice received a single i.v. dose of 1 mg of pCons, dissolved in saline, as reported previously (29, 31, 36) for tolerance induction. For immunophenotyping of regulatory B cells, female 35-wk-old BWF1 mice were used and injected with pCons (1 mg *i.v.*). After 3 days, blood was obtained, RBC lysed, and cells were stained with CD19, CD1d and CD5 antibodies and FACS performed. Control mice received either a similar amount of pNeg (negative control peptide) or saline.

### Cell Isolation, Preparation, Immunophenotyping, and Flow cytometry

Spleen cells were isolated ~1 week after administration of the pCons peptide from tolerized, saline-treated, or naïve BWF1 mice. Single cell suspensions of splenocytes were prepared by passing cells through cell strainers (Fisher). ACK lysing buffer, (Sigma, St Louis, MO, USA) was used to lyse red blood cells. Cells were washed and re-suspended in RPMI complete media. Cell subsets were further enriched following incubation with anti-CD4 (L3T4), anti-B (CD45R/B220), anti-CD8 (CD8a Ly-2), anti-NK1.1 (CD95b), anti-DX5, anti-CD11C, and anti-Gr-1 microbeads from Miltenyi Biotech (Auburn, CA, USA). Purity of cells was determined to be more than 90% pure as assessed by flow cytometry (FACS). For immunophenotyping, isolated cells were washed with FACS buffer and 1–2 million cells were used for surface staining. Before staining, cells were incubated with rat anti-mouse CD16/CD32 (FC III/II receptor) Ab to block nonspecific binding.

For regulatory B cell immunophenotyping, female 35 wk-old BWF1 mice were treated with pCons 1 mg *i.v*. and blood was obtained after (3-5 days). Splenocytes were depleted of red blood cells (RBCs) and then stained with CD19, CD1d, and CD5 antibodies for FACS analysis. Antibodies for cell surface staining and isotype controls were from BD Biosciences, BD Pharmingen, eBiosciences, or BioLegend. CD4 (L3T4), CD25 (PC61.5) and CTLA-4 (UC10-4F10-11) staining was performed with antibodies from BD Pharmingen. FoxP3 (FJK-16s) staining was performed with an eBiosciences intracellular kit. Data were collected using FACSCalibur (BD Biosciences) and analyzed by BD Cell Quest software (Becton-Dickinson, Mountain View, CA) or FCS De Nova software (Thornhill, Ontario, Canada).

### Western Blot Analysis

Western blot analyses were performed as described earlier (37). In brief, cell lysates were prepared from the naïve and tolerized B cells from the splenocytes of naïve and pCons-treated BWF1 mice. Cells were lysed with RIPA buffer (150 nM NaCl, 1.0% NP-40, 0.5% sodium deoxycholate, 0.1% SDS, 10 mM Tris, pH 7.3) supplemented with Protease Arrest protease inhibitor cocktail solution (G Biosciences, Maryland Heights, MO, USA). Protein was measured from each sample using the Bradford assay (Bio-Rad Laboratories, Hercules, CA, USA) and an equal amount of protein was loaded in each well. The lysates were resolved on a 4–12% NuPage gel (Invitrogen, Carlsbad, CA, USA) in reducing conditions. Proteins were electro-transferred onto a polyvinylidene fluoride membrane (Invitrogen). The membranes were blocked with 3% BSA and immunoblotted with a specific antibody, bcl2 (50E3), (1:200 dilution; Cell Signaling Technology, Inc.) or β-actin (1:100 000 dilution; Sigma, Inc.). Following washing, the membranes were incubated in secondary antibodies (1:2500 dilution; Santa Cruz Inc, Santa Cruz, CA, USA). All blocking, incubation and washing steps were performed in TBST (TBS and 0.1% Tween 20). Proteins were visualized using ECL (GE Healthcare, Buckinghamshire, UK).

### RNA Isolation and Real-Time PCR

Total cellular RNA was isolated from purified cell subsets or total splenocytes from saline-treated or pCons-tolerized BWF1 mice with TRIzol (Invitrogen, Carlsbad, CA, USA) as per manufacturer’s protocols. Real-time PCR was performed as described earlier (29, 33–35). Each experimental group consists of the pooled spleen cells of 3–4 mice from each group. 100 ng of total RNA was used with one-step RT-PCR reagents (Applied Biosystems, Foster City, CA, USA). Quantitative real-time reverse transcription was performed using TaqMan technology on an ABI Prism 7900 HT Sequence Detection System (Applied Biosystems). Primers and probes of *IFI205*, *GATA1*, *Ptdss2*, *TNFip*2, *FoxP3* and *GAPDH* were obtained from Applied Biosystems, Foster City, CA, USA. The oligonucleotide sequences used for the primers and TaqMan probes (Applied Biosystem, Foster City, CA) are described (29, 33–35). GAPDH was used as an endogenous control in each experimental set.

### Cell Culture and Measurement of Anti-DNA Antibodies

Assays were performed to measure anti-DNA Ab as described earlier (29, 31, 34, 35, 37). For optimal Ab production, B cells (1x10^5^ cells) from keep old (40-50-wk-old) naïve BWF1 females with 3+ proteinuria or higher, CD4^+^CD25^-^ T cells (1x10^6^) from young 10–12-wk-old naive BWF1 females without proteinuria, naïve CD8^+^ T cells (1x10^6^), and irradiated APC (1x10^5^) cells were isolated and cultured with granulocytes or B cells (1x10^6^) from tolerized mice or controls. Cell cultures were performed in RPMI 1640 supplemented with L-glutamine (2 mM), penicillin (100 units/ml), streptomycin (0.1 mg/ml), 2-mercaptoethanol (Gibco) and 10% fetal bovine serum (FBS). For tolerized B cells analysis, we cultured, as indicated above pCons-tolerized B cells (1x 10^6^) with naïve CD4^+^CD25^-^ T cells (1x 10^6^), naïve B (1x 10^5^) and/or naïve CD8^+^ T cells (1x10^6^) cells. After 72-96 hours, culture supernatants were obtained and anti-DNA IgG was measured by ELISA.

### Statistical Analyses

Data were analyzed using Prism 4.0 (GraphPad Software, San Diego, CA). Comparisons were performed using paired one- or two-tailed test. Nonparametric testing among more than two groups was performed by one-way ANOVA. Results are expressed as mean ± SEM. p<0.05 was considered significant.

## Results

### pCons-Induced Tolerized B Cells and Granulocytes Suppressed Anti-DNA Ab Production by BWF1 Cells

**Figure 1:**
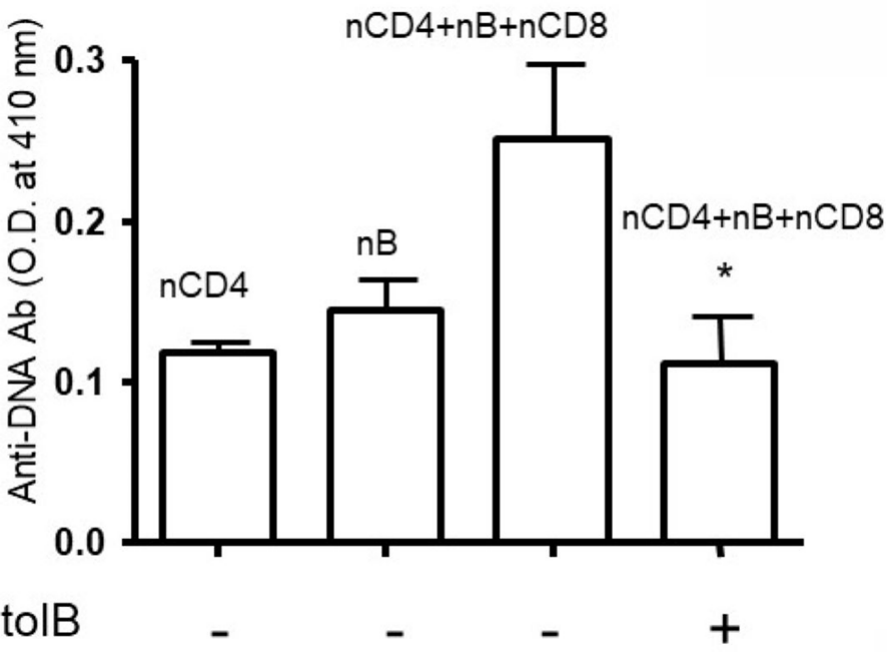
Anti-DNA Ab was significantly decreased in the presence of tolerized B cells. Naïve CD4^+^ T cells, CD8+ T cells and CD45R/B220^+^B cells were isolated from BWF1 mice spleen cells using microbeads from Miltenyi Biotech (Auburn, CA, USA). Cells were cultured in RPMI 1640 supplemented with L-glutamine (2 mM), penicillin (100 units/ml), streptomycin (0.1 mg/ml), 2-mercaptoethanol (Gibco) and 10% fetal bovine serum (FBS). Cells were co-cultured in the presence of tolerized B cells (1x10^6^ cells). Different immune cell subsets (naïve B cells, 1x10^5^ cells from old nephritic mice; CD4^+^CD25^-^ T cells, 1x10^6^; naïve CD8^+^ T cells, 1x10^5^) were isolated from splenocytes and cultured with tolerized B cells (1x10^6^ cells). After the 72-96 hours range, culture supernatants were obtained. Anti-DNA Ab levels were measured from culture supernatants by ELISA. *p < 0.05.

**Figure 2:**
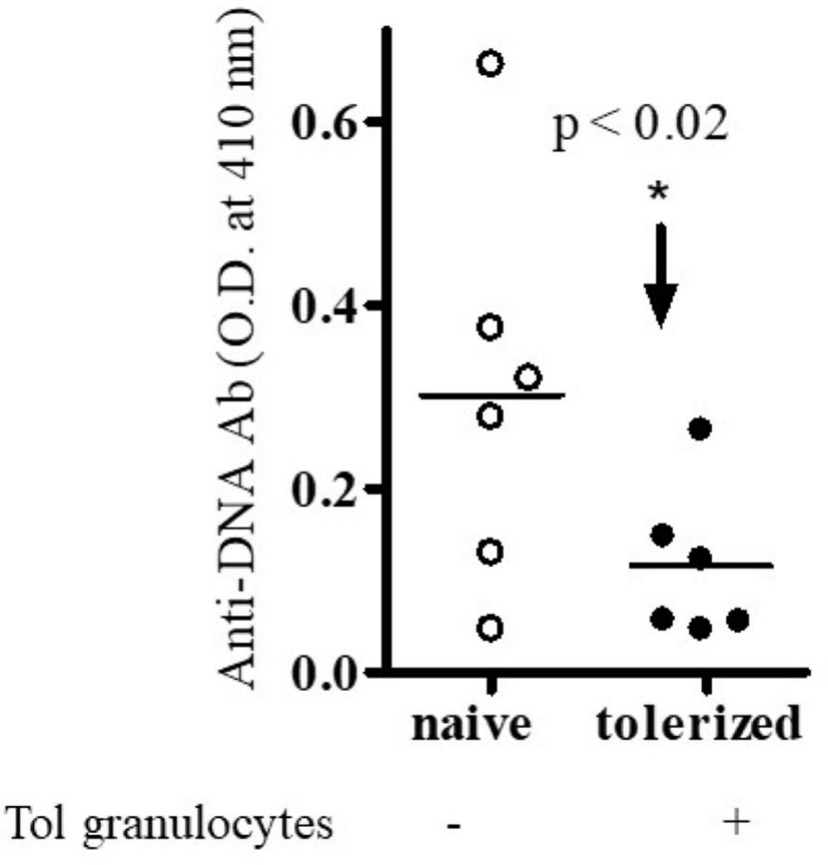
Anti-DNA Ab was significantly decreased in the presence of tolerized PMNs-granulocytes. Different immune cell subsets (naïve B cells, 1x 10^5^ cells from old nephritic mice; CD4^+^CD25^-^ T cells, 1x10^6^; naïve CD8^+^ T cells, 1x10^5^) were isolated and cultured with tolerized granulocytes (GR, 1x10^6^). Cell subsets were isolated from total spleen cells of BWF1 mice. Cells were cultured in RPMI 1640 supplemented with L-glutamine (2 mM), penicillin (100 units/ml), streptomycin (0.1 mg/ml), 2-mercaptoethanol (Gibco) and 10% fetal bovine serum (FBS). After the 72- 96 hours range, culture supernatants were obtained. Anti-DNA Ab levels were measured from culture supernatants by ELISA. *p < 0.05.

To our knowledge, no studies have been performed to address the role of regulatory B cells and granulocytes in the immune tolerance and BWF1 lupus. To address this, we harvested B cells and granulocytes from the spleens of naïve and tolerized BWF1 mice 7 days after the induction of tolerance (peptide treatment 1 mg *i.v*. once a week). We used *in vitro* assays to test the effects of each cell type on anti-DNA Ab production with the addition of naïve CD4^+^ helper cells (CD4^+^CD25^-^ T cells) plus naïve B cells (from old BWF1 nephritic mice) and naïve CD8^+^ T cells to each cultured subset either separately or in combination with tolerized B cells (B_regs_) and granulocytes. Anti-DNA Ab was analyzed as described previously (29, 31, 34, 35, 37). Briefly, purified populations of different spleen effector cell subsets including B cells, naïve CD4^+^T cells (CD4^+^CD25^-^Tcells), naïve CD8^+^ T cells, and tolerized granulocytes and B cells were harvested (using the appropriate Miltenyi Biotec microbeads via AutoMACS) one week after pCons treatment of BWF1 mice. Naïve CD4^+^ T cells from young mice (10-12-weeks-old) and B cells from old nephritic BWF1 mice (40-50-weeks-old with 3+ proteinuria) were co-cultured in complete medium with tolerized/regulatory B cells and granulocytes and other effector cell types such as (naïve CD8^+^ T cells) from spleens of tolerized mice. Our previous cell-dose response study with CD8^+^ Treg showed that 1×10^6^ cells are optimum in mixed cell culture experiments (29) therefore, we used the same number of cells in these experiments. In addition previously, we had found that a ratio of B cells to helper T cells (CD4^+^CD25^-^T cells) to regulatory/suppressor T cells of 1:10:10 are needed to observe optimal suppression of anti-DNA antibodies (29, 32, 35). Therefore, we used this same ratio with each effector cell type. We found that both tolerized B cells and granulocytes suppressed the production of anti-DNA Ab (**Figures 1** and **2**). Although we did not determine whether this suppressive effect was direct or indirect on autoreactive B cells through CD4^+^ or CD8^+^ T cells or by synergistic effect by those cells with tolerized B cells and granulocytes, this data clearly suggests that pCons-induced regulatory B cells and granulocytes suppress the anti-DNA Ab production and thus play a significant role in autoimmunity.

### Microarray Analysis Showed Altered Regulation of Genes in Non-T Cells

Since our previous published microarray data showed that pCons treatment induces major changes in white blood cells (WBC) subsets of BWF1 spleen cells [448 genes differentially-regulated in whole splenocytes of tolerized compared to control mice (33)], we were interested to see the potential role of regulatory B cells and granulocytes. Thus, in this report, we characterized expression of selected genes (highly upregulated) in different cell populations in non-T cells (tolerized B cells and tolerized granulocytes cells) and further tested the ability of these cell subsets to suppress production of anti-DNA Ab in lupus. Our data suggests that cell types other than T cells may play major roles in this model of immune tolerance.

### B cells and Granulocytes Produced Significantly Increased/Decreased Amounts of mRNA for Several Genes of Interest Including Interferon Genes After pCons Treatment in BWF1 Lupus Mice

**Figure 3:**
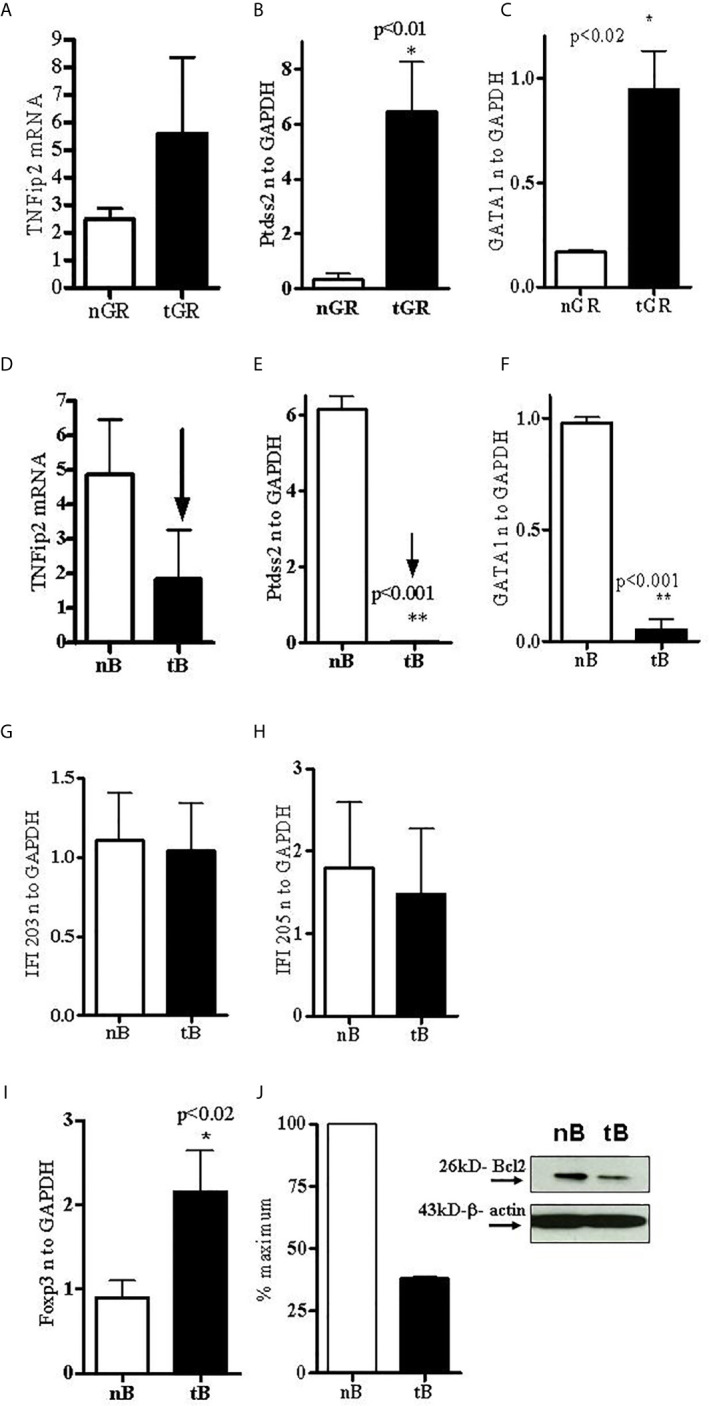
Tolerized B cells have reduced IFNs gene mRNA and Bcl2 protein level and increased FoxP3 mRNA expression. RNA was isolated from naïve and tolerized B cells and granulocytes. Real time PCR was performed with 100 ng of RNA with gene specific primers and probes. Data was normalized with GAPDH mRNA levels. *p < 0.05. *TNFAIP2*, *Ptdss2*, and *GATA1* mRNA was increased **(A–C)** in tolerized granulocytes (GR) but reduced in tolerized B cells **(D–F)**. *IFI203* and *IFI205* was decreased in tolerized B cells **(G, H)**. **(I)** *FoxP3* expression was increased in tolerized B cells. **(J)** Quantification of Western blot analysis of Bcl2 protein levels in cell lysates from naïve and tolerized sorted B (CD45R/B220) cells.

To address the role of tolerized non-T cell subsets after pCons treatment in BWF1 mice, B cells and granulocytes were obtained from the spleen of BWF1 mice 1 weeks after pCons treatment. RNA was isolated from these cell subsets and real-time PCR was performed as described earlier (33). Real-time PCR analyses indicate that *TNFAIP2 (*Tumor necrosis factor alpha-induced protein 2) was increased ~2-fold (**Figure 3A**), and *Ptdss2 (*Phosphatidylserine synthase 2 *and GATA1 (*GATA-binding factor 1) *mRNA were upregulated more than 10-fold in tolerized granulocytes compared to naïve granulocytes* (**Figures 3B, C** **)**. In contrast, the mRNA of all the above genes (**Figures 3D–F**) and those of IFN-induced genes including *IFI203* and *IFI205* (**Figures 3G, H**) were down regulated in tolerized B cells. Although, the decreased level of *IFI203 and IFI205* did not reach to the significance. However, other IFNs genes were significantly decreased. Thus, this data displays dynamic interplay and suggests that pCons has differential effects on different interferon genes in our tolerance model. Collectively these data demonstrate that pCons treatment modify the interferon’s gene signature differentially in tolerized B cells and granulocytes.

### pCons-Tolerized B Cells From Lupus Mice Have Increased FoxP3 mRNA and Bcl2 Protein Levels

The transcription factor forkhead box P3 (FoxP3), also known as scurfin, plays an important role in the maintenance of immunological homeostasis and restoration of self-tolerance. Dysfunction and mutations of the *FoxP3* gene causes immunodysregulation polyendocrinopathy enteropathy X-linked (or IPEX) syndrome. *FoxP3* also participates in maintaining the immune system response (38) and in the development and function of regulatory T cells (39–42). In the present study, we evaluated the expression of *FoxP3* in pCons-tolerized B cells of lupus (BWF1 mice) compared with naïve B cells. Surprisingly, we found significantly increased expression of *FoxP3* in tolerized B cells compared to naïve B cells (**Figure 3I**). Next, we investigated the protein expression of *bcl2* from cell lysates of tolerized B cells and naïve B cells with Western blot assay. We found that tolerized B cells have decreased levels of Bcl2 protein compared to naïve B cells (**Figure 3J**). Bcl-2 regulates cell death (apoptosis) by promoting or inhibiting apoptosis (43, 44). We have shown previously that CD4^+^ and CD8^+^ T cells from tolerized mice have significantly reduced apoptosis. Thus our data suggest that pCons tolerance may also affect apoptosis of B cells in our tolerance model and may play a significant role in survival of these cells by regulating immune tolerance.

### pCons Treatment Induced and Modified the Cell Surface Expression Markers for Regulatory B Cells

**Figure 4:**
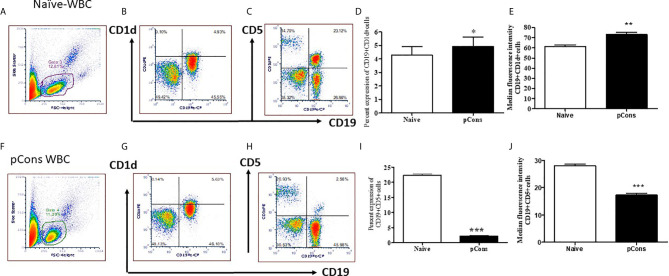
pCons treatment modified the cell surface expression markers for regulatory B cells. Female 35-wk old BWF1 mice were treated with pCons (1 mg i.v.). After 3 days, blood was obtained, RBC lysed, and cells were stained with CD19, CD1d and CD5 antibodies and FACS performed. Representative live cell gating strategy **(A, F)** and FACS analysis of CD1d **(B, G)** and CD5 **(C, H)** expression levels from representative naïve **(A–C)** and pCons treated **(F–H)** mice. Percent expression of CD19^+^ CD1d^+^ cells in naïve vs pCons treated mice **(D)**. Percent expression of CD19^+^CD5^+^cells in naïve vs pCons treated mice **(I)**. Quantification of Median Fluorescent Intensity of naïve and pCons-treated mice for CD1d **(E)** (from B and G Gate 3 and 4) and CD5 **(J)** (from C and H Gate 3 and 4). CD1d increased and CD5 cells decreased with pCons treatment. *p < 0.05, **p < 0.001, ***p < 0.0001.

Our previous study showed that pCons treatment induces both CD4^+^ and CD8^+^ regulatory T cells (29, 32, 36). Based on these data and our gene expression study (33), we hypothesize that pCons treatment may induce suppressor/regulatory B cells (B_regs_) and granulocyte cells *with* the potential to suppress the proliferation of naïve CD4^+^CD25^-^ cells and naïve B cells as well as the production of anti-DNA Ab. To address this, we isolated spleen cells from female BWF1 mice after one week of pCons treatment (1 mg *i.v*) and performed immunophenotyping studies with flow cytometry from naïve and pCons-treated mice (**Figures 4A, F** live gating scheme). We found that pCons treatment of BWF1 mice increases percent expression of CD19^+^CD1d^+^ regulatory B cells including median fluorescence intensity (**Figures 4B, G, D, E**). This is an important finding because two previous studies have revealed similar phenotype of B_regs_ in SLE patients (45, 46). Further, we found that pCons treatment reduces the percent expression of CD19^+^CD5^+^ B cells (**Figures 4C, H, I**). The median fluorescence intensity (MFI) of CD19^+^ CD5^+^ cells were significantly decreased in pCons treated mice (**Figure 4J**). These data show that pCons treatment modified the B cells expression markers CD1d and CD5, and since we have also shown that CD4^+^ and CD8^+^ T cells from tolerized mice suppress autoreactive B cells and could account for their reduced numbers, this suggests that pCons treatment induces regulatory B cells.

### pCons Treatment Increased CD4^+^FoxP3^+^ Regulatory T Cells and Significantly Reduced Percent Expression and Median Fluorescence Intensity of CTLA-4 (Cytotoxic T-Lymphocyte-Associated Proten-4) in CD8^+^ T Cells of BWF1 Lupus Mice

**Figure 5:**
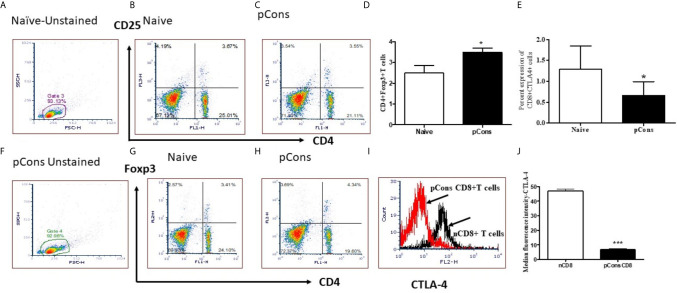
pCons treatment increased CD4^+^FoxP3^+^ regulatory T cells and significantly reduced Median Fluorescence Intensity of CTLA-4 (Cytotoxic T-lymphocyte-Associated Proten-4) in CD8^+^ T cells of BWF1 lupus mice. Female 12-20 wk-old BWF1 mice were treated with pCons (1mg i.v.). After 1-2 weeks, splenocytes were obtained, RBC lysed, and cells were stained with CD4, CD8, CD25, CTLA-4 and FoxP3 antibodies and FACS performed. 10,000 minimum cells were gated. Representative gating strategy **(A, F)** and FACS analyses of CD4^+^CD25^+^ **(B, C)**, CD4^+^FoxP3^+^ **(G, H)** and cumulative two-three experiments data for CD4^+^FoxP3^+^ cells **(D)** are shown. Cumulative data of CD8^+^ CTLA-4^+^ T cells experiments (two-three) is shown **(E)**. CTLA-4 staining (MFI) on CD8^+^ T cells is shown **(I, J)**. CD4^+^FoxP3^+^T cells are significantly increased. CTLA-4 MFI is significantly decreased. *p < 0.05, **p < 0.001, ***p < 0.0001.

We were interested to see whether pCons treatment induces regulatory T cells and whether it affects CTLA-4 expression. CTLA-4 plays an important role in immune tolerance and T-cell activation. We found that pCons treatment significantly increased the number of CD4^+^FoxP3^+^ T cells in BWF1 mice compared to naïve and/or saline-treated mice (**Figures 5A–D, F–H**). We also measured the CTLA-4 expression on T cells (CD8^+^T cells) and found that percent expression of CTLA-4 was significantly decreased in pCons treated mice (**Figure 5E**) Further, we found that pCons treatment significantly reduced the median fluorescence intensity (MFI) of CTLA-4 expression in CD8^+^ T cells compared to naïve mice (**Figures 5I, J**). Thus, the data shows the immunomodulatory role of pCons in BWF1 mice. However, future study is warranted to pinpoint the exact mechanism of pCons activity in Lupus.

## Discussion

In the present study, we have added to previous work showing that pCons induces CD8^+^ and CD4^+^ suppressive cells and shown that B cells and granulocytes from tolerized mice suppress anti-DNA Ab production *in vitro*. Several suppressive mechanisms/factors may be involved including IL-10, TGFβ, IL-35, and combinations of TLR9, CD40, and/or B cell receptor (BCR) and engagement of CD80/CD86 on B_regs_ (45, 47). pCons treatment significantly increased the number of CD4^+^FoxP3^+^ T cells. In earlier studies, we showed that these FoxP3^+^ T cells (both CD4^+^ and CD8^+^ T_reg)_ suppressed autoimmunity *in vivo* and anti-DNA production *in vitro* (29, 32, 36). Immune tolerance induced by pCons prolonged survival of BWF1 lupus mice (NZB/NZW) F1 and delayed the appearance of glomerulonephritis (29, 31, 35). The pCons-induced regulatory T cells suppressed proliferation of naïve CD4^+^ T cells and naïve CD19^+^/B220^+^ B cells and the production of anti-DNA antibodies (29, 32, 34–36). T cell suppressive capacity correlated with modulation of Mitogen-Activated Protein kinase (p38 MAP kinase) activity and FoxP3 expression in CD4^+^ Tregs (48). In the current study we found that CTLA-4 median fluorescence intensity was significantly decreased in the CD8^+^ T cells of pCons-treated BWF1 mice. This is a significant finding as CTLA-4 is involved in negative signaling and plays a pivotal inhibitory role in T cell anergy and prevention of autoimmunity. In addition, recent studies show that CTLA-4 controls follicular helper T cells and regulatory T cells, thereby controlling the B cells responses and humoral immunity (49–51). CTLA-4 also downregulates CD80 and CD86 on antigen presenting cells (APC); thus, altering the level of CD28 engagement on follicular helper T cells (51). However, its precise mechanism of action has not been fully resolved. Recently abnormal CTLA-4 gene polymorphisms and function has been reported in SLE patients (52, 53).

For the first time to our knowledge, in this system we found that B cells and granulocytes also can be “tolerized” and subsequently function as regulatory/suppressor cells to prevent production of autoantibodies. In the current experiments, we used whole tolerized B cells and granulocytes for the suppression assay. We acknowledge the next step is to test the specific B_regs_ subsets to determine cell specificity and their mode of action and mechanism. Therefore, detailed molecular and cellular mechanisms of regulatory B cells and granulocytes are not completely clear and future study will be required to address this shortcoming.

Regulatory B cells and regulatory granulocytes are not well characterized in this SLE tolerance model and this study provided first novel mechanistic insight. We showed that 1) pCons tolerance altered expression of several candidate genes (see below) including interferon genes in tolerized B cells and granulocytes compared to naïve cells; 2) pCons tolerance modified the cell surface expression of regulatory B cells (and/or deleted the CD19^+^CD5^+^ subset); 3) pCons tolerance increased the percent expression of (CD19^+^CD1d^+^) cells; and 4) pCons-tolerized B cells and granulocytes significantly reduced the production of anti-DNA antibody in cell culture experiments of lupus mouse cells.

pCons tolerance has been shown to affect various genes and markers, cell surface molecules, cytokines and different cell types including regulatory T cells (both CD4 and CD8) (29–35, 37). In the present study, we showed that pCons induced B cells enriched in markers identifying suppressor B cells and these cells have significantly reduced interferon-induced genes (*IFN*) such as, *ptdss2*, *GATA1*, and T*NFip2* (**Figure 3**) compared to naïve mice. In contrast, we also found that these genes were significantly increased in tolerized granulocytes, with the exception of *TNFip2* which was upregulated but did not reach the significance level (**Figure 3**) demonstrating differential effect of pCons. Thus, our data indicate dynamic interplay of these genes or their gene products in different immune cell subsets in our pCons-induced tolerance model. How this interplay affects the overall immune response in lupus mice is not clear. However, recent studies have shown the importance of interferon genes in lupus (54–58). Lupus is characterized by the dysregulation of both the innate and the adaptive immune systems. An increased expression of type I IFN-regulated genes, termed IFN signature, has been reported in the majority of patients with SLE (59–61). In agreement with our findings, another study found that a tolerogenic peptide of the light chain complementarity-determining region 1 (hCDR1) down-regulates the expression of interferon-alpha (IFN-α) in murine and human SLE (62). IFN-α plays a major role in SLE pathogenesis and the levels of IFN-α were increased and correlated with SLE disease activity in the sera of mice and humans (63–65). Administration of exogenous IFN-α leads to worsening of disease in various mouse models (66). Type 1 IFN contributes to loss of tolerance and increases production of autoantibodies (67), induces differentiation of monocytes to myeloid-derived dendritic cells (mDC) (56), and plays a vital role in the activation of autoreactive T and B cells (68). Activation of TLR7 and TLR9 is thought to be central to induction of the type 1 IFN response (69, 70). Indeed, a recent therapeutic option in patients with lupus is through inhibition of type IFN–α and several recent clinical trial data suggest therapeutic benefit (71–73). Thus, our findings that pCons tolerance reduces the IFN genes in our lupus model has direct clinical and translational significance.

We have shown earlier that pCons peptide delayed the onset of autoimmunity in lupus mouse model by inducing immune tolerance and up-regulating FoxP3 in T cells which are suppressive (31, 34, 74). Other studies have reported that peptides from CDRs of pathogenic anti-DNA Ab could also prevent autoantibody production and down-regulate autoreactive T cell responses (75, 76). Similar to our results, these studies showed that a peptide derived from the CDR1 of a human anti-DNA Ab (hCDR1) could ameliorate lupus by inducing T_regs_ and suppressing the activation of autoreactive T cells through mechanisms including downregulation of transcription factors responsible for negative regulation of T-cell activation in lupus animal models (62, 76–79). Furthermore, clinical trial data has indicated safety and efficacy of hCDR1 (edratide) in SLE patients (80).

We demonstrated with flow cytometry (FACS) immunophenotyping that after pCons treatment the CD19^+^CD1d^+^ regulatory B cell subset was significantly increased in BWF1 lupus mice compared to naïve mice (**Figures 4B, G, D**). However, the CD19^+^CD5^+^ B cell subset was significantly decreased (**Figures 4C, H, I**). This is a significant finding and the next step will be to decipher the mechanisms with future functional studies including testing B cells from anti-CD5 treated control mice. In agreement with our experiments, a previous study showed that anti-CD5 therapy decreases severity of established disease in collagen-induced arthritis in DBA/1 mice (81). Thus, our data with pCons therapy has clinical and therapeutic relevance in peptide induced immune tolerance. We also found that tolerized B cells have significant increased *FoxP3* mRNA. Another study reported that the expansion of CD25^+hi^CD5^+^ and FoxP3^+^ regulatory B cells is associated with SLE disease activity in humans (82). Similarly, the presence of FoxP3^+^ CD19^+^CD5^+^ B cells in human peripheral blood mononuclear cells has also been reported (83). The diverse suppressive mechanisms of these regulatory B cells are through IL-10, TGFβ, and IL-35. Previously we have demonstrated that pCons-induced splenocytes have significantly increased amount of TGFβ, smad2, and smad3 expression and tolerized total CD8^+^ T cells have increased amount of IL-10 (37). Although, we did not measure the expression of IL-10, TGFβ and IL-35 in the regulatory B cells in our model, it is tempting to speculate that these molecules will play important role in our system based on our previous data. Thus, our findings may suggest that pCons tolerance promotes tolerized B cells that can suppress the autoimmune responses. Similar to our study, hCDR1 tolerance has effects on B cell activating factor (BAFF) and B-cell CD74 macrophage inhibitory factor in murine lupus (84, 85). The reduced levels of BAFF correlated with reduced rate of maturation and differentiation of B cells and decrease in integrin expression. Recent studies provided further evidence of targeting of BAFF/BLys and APRIL in the management of lupus (86–88); and another study reported the effect of hCDR1 on IL-7 and apoptosis (89) and showed the rate of apoptosis is reduced with hCDR1 treatment in lupus mice. Bcl2 and Bcl-_XL_ levels were further reduced, and this was associated with reduced activation of T and B cells (90). We demonstrated earlier that pCons-induced CD8^+^ T_regs_ are resistant to apoptosis (29, 34). In the present study, we found that bcl2 protein level was significantly decreased in tolerized B cells compared to naïve B cells thus affecting the survival of these cells in BWF1 mice. This is in agreement with another study that revealed increased expression of Bcl2 leads to development of SLE like symptoms in Bcl2 transgenic mice (91). Thus, altogether, our data suggests that pCons’ effect on tolerized B cells and down-regulation of IFNs and bcl2 may play overall therapeutic beneficial effects in our tolerance model.

## Data Availability Statement

The raw data supporting the conclusions of this article will be made available by the authors, without undue reservation.

## Ethics Statement 

The animal study was reviewed and approved by UCLA Animal Research Committee (ARC).

## Author Contributions

RPS contributed to the experimental design, obtaining funding, conducting experiments, analyzing data, preparing figures, and writing of the manuscript. BHH contributed to funding and editing of the manuscript. DSB contributed to figure and manuscript editing. All authors contributed to the article and approved the submitted version.

## Funding

This work was supported by the NIH grants AR54034, AI 083894, AI65645 to RPS, UCLA Senate Core Grant to BHH and RPS, and UCLA Oppenheimer Clinical Seed Grant and American Autoimmune Related Disease Association grant to RPS.

## Conflict of Interest

BHH and RPS have a patent through the University of California, Los Angeles for the use of pCons as an immune modulator in systemic lupus erythematosus.

The remaining author declares that the research was conducted in the absence of any commercial or financial relationships that could be construed as a potential conflict of interest.
